# Analyses of variant human papillomavirus type-16 E5 proteins for their ability to induce mitogenesis of murine fibroblasts

**DOI:** 10.1186/1475-2867-6-19

**Published:** 2006-08-09

**Authors:** Rahul Nath, Christine A Mant, Barbara Kell, John Cason, Jon M Bible

**Affiliations:** 1 Department of Infectious Diseases, Second Floor New Guy's House, Guy's Hospital, Guy's, King's College and St Thomas' School of Medicine, King's College London, London SE19RT, UK

## Abstract

**Background:**

Human papillomavirus type 16 (HPV-16) E5 protein co-operates with epidermal growth factor to stimulate mitogenesis of murine fibroblasts. Currently, little is known about which viral amino acids are involved in this process. Using sequence variants of HPV-16 E5 we have investigated their effects upon E5 transcription, cell-cycling and cell-growth of murine fibroblasts.

**Results:**

We demonstrate that: (i) introduction of Thr^64 ^into the reference E5 sequence of HPV-16 abrogates mitogenic activity: both were poorly transcribed in NIH-3T3 cells; (ii) substitution of Leu^44^Val^65 ^or, Thr^37^Leu^44^Val^65 ^into the HPV-16 E5 reference backbone resulted in high transcription in NIH-3T3 cells, enhanced cell-cycle progression and high cell-growth; and, (iii) inclusion of Tyr^8 ^into the Leu^44^Val^65 ^backbone inhibited E5 induced cell-growth and repression of p21 expression, despite high transcription levels.

**Conclusion:**

The effects of HPV-16 E5 variants upon mitosis help to explain why Leu^44^Val^65 ^HPV-16 E5 variants are most prevalent in 'wild' pathogenic viral populations in the UK.

## Background

**Figure 1:**
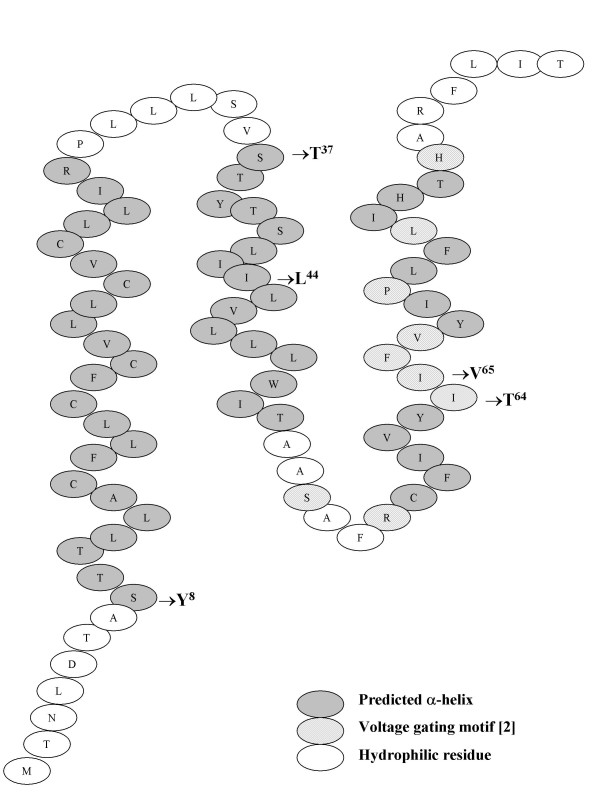
**Putative structure of HPV-16 E5**. Cartoon representation of the averaged results of multiple secondary structure predictions of reference sequence of HPV-16 E5 protein [25, 26] (*e.g*. using programmes at : data not shown) showing the position of the predicted α-helices, the proposed voltage gating motif, as well as the amino-acid mutations of the natural variants studied.

A causal association between high-risk human papillomaviruses (HPV) infection – particularly HPV-16 – and cervical cancer has been established. HPV-16 E5 is a minor oncoprotein comprising of 83 amino acids and *in silica *predictions suggest it comprises of 3 anchor-like α-helices (residues 8–30, 37–52 and 58–76): with only the first being sufficient to span a lipid bilayer (Figure 1). A region within the second helix (residues 41–54) may be the binding site for the pore sub-unit of 16 KDa ATPase [1], though others claim it is located between residues 54–78 [2]. HPV-16 E5 is believed to act in the early stages of the oncogenic process [3-6] and is membrane-associated, occurring in the Golgi apparatus and endoplasmic reticulum [7].

HPV-16 E5 acts co-operatively with epidermal growth factor (EGF) to stimulate mitosis. Whilst E5 may, or may not, bind directly to the EGF-receptor (EGFr) [8,9], it was initially believed to impair acidification of endosomes *via *interaction with 16 KDa ATPase [10,11] and thereby promote recycling of EGFr to the cell-surface [10]. Others have suggested: that E5 perturbs EGFr trafficking from early to late endocytic structures rather than influencing acidification [12]; or, that E5 uses 16 KDa ATPase as a chaperone to enter the Golgi [2,13].

**Figure 2:**
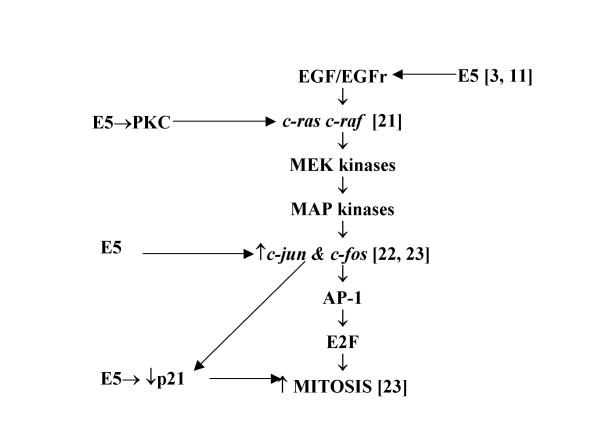
**Points at which HPV-16 E5 affects the epidermal growth factor signal transduction pathway**. EGF: epidermal growth factor; EGFr: epidermal growth factor receptor; PKC: protein kinase C; AP-1: activator protein 1.

Whatever the initial processes, HPV-16 E5 stimulates *c-ras*, causing *c-raf *to attach to plasma membranes, activating enzyme cascades through the MEK and MAP kinases, which in turn migrate to the nucleus to phosphorylate *c-fos *transcription factors [14-17]. Ultimately, this permits assembly of activator protein-1 heterodimers from *c-fos *and *c-jun *and stimulation of mitosis [18-20]. E5 can interdict this pathway *via*: (i) the induction of protein-kinase C which activates *c-raf *[21]; (ii) initiation of *c-jun *and *c-fos *and *junB *transcription [22,23]; and (iii), repression of p21 expression (a cyclin-dependant kinase inhibitor which causes pocket-protein phosphorylation, release of E2F and – *via de novo *synthesis of cyclins A, B and E – mitosis [23]: Figure 2).

We have previously described HPV-16 E5 variants which encode novel E5 protein sequences [24]. Here we conjectured that these natural E5 protein variants may have differing mitogenic properties and that the amino acids involved in this process might be discernable. This proposal was based upon evidence that certain HPV-16 E5 variants are more prevalent than others in wild viral populations in our locality. We detected marked differences in the ability of individual HPV-16 E5 variants to induce transcription in stably transfected long-term NIH-3T3 cell lines, changes in cell-cycle profiles and cell-growth. Some variants were more mitogenic than the reference isolate of HPV-16 E5: others which were poorly mitogenic as a result of either amino acid changes or, low transcriptional efficiencies. Interestingly, those HPV-16 E5 variants most frequently detected in our local population (RFLP pattern 2) – and most commonly associated with cervical lesions – were those which had the greatest mitogenic activity *in vitro*.

## Results

### HPV E5 constructs

HPV-16 E5 variants: Leu^44^Val^65 ^(AJ244882); Thr^37^Leu^44^Val^65 ^**(**AJ44863); Thr^64^**(**AJ244840) [24]; Tyr^8^Leu^44^Val^65^, (AJ24481); the HPV-16 reference E5 sequence [25,26] and HPV-6b E5 were amplified and cloned into *pc*DNA3.1*Myc*-His. For each HPV-16 E5 variant, a control construct, containing a TAA 'stop' codon (at codon position three) was also cloned into *pc*DNA3.1*Myc*-His.

**Figure 3:**
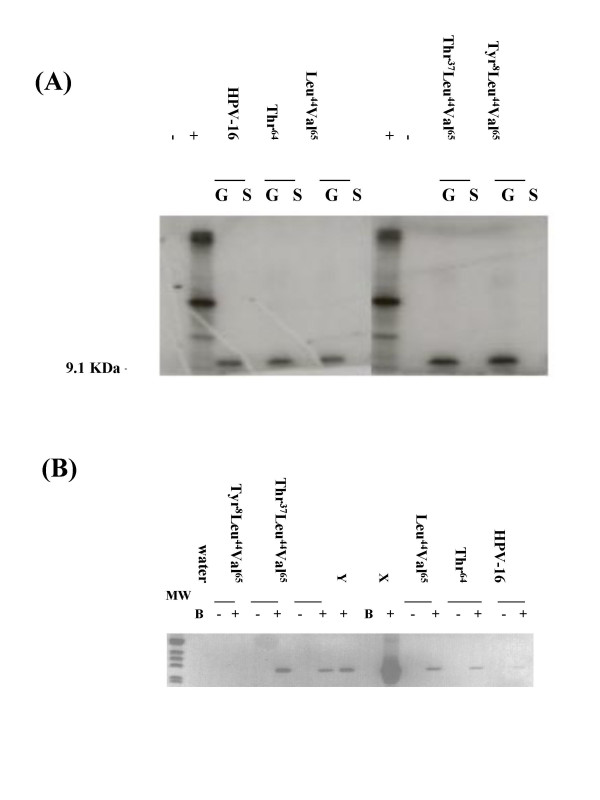
**Expression of E5 protein *in vitro *and cell line mRNA**. **(A) **Autoradiographs of PAGE gels containing *in vitro *wheat germ protein translation products of HPV-16 E5 variants (G) and 'stop' codon controls (S), demonstrating ^35^S-labelled 9.1 kDa products in the former, but not the latter. +: positive (luciferase), and -: negative kit controls. X: 1 ng HPV-16 RNA ; Y: 0.1 ng HPV-16 RNA. **(B) **Expression of E5 mRNA detected by RT-PCR in transfected cell-lines analysed in the presence (+) or absence of reverse transcriptase. B: blank.

All constructs and 'stop' controls had the correct DNA sequences after cloning (data not shown). T7 'run-off' transcripts were prepared and translated in cell-free wheat-germ expression assays spiked with ^35^S-labelled cysteine (all have 4 cysteines). Autoradiographs of polyacrylamide gel elecprophoresis (PAGE) gels revealed equivalent levels of *in vitro *translation for all HPV-16 variants, but no evidence of protein products from the equivalent 'stop' controls (Figure 3A), confirming the fidelity of TAA ('stop') codons. However, different HPV-16 E5 variants were transcribed at different levels in stably-transfected (G418-selected) NIH-3T3 cell-lines, with Tyr^8^Leu^44^Val^65 ^being expressed at higher level than either Thr^37^Leu^44^Val^65^, or Leu^44^Val^65^, whilst the Thr^64 ^variant and reference sequence were transcribed at low level (Figure 3B). To assist data interpretation, cell-lines for all 'stop' constructs were pooled prior to subsequent experiments.

### Co-operation between HPV-16 E5 and EGF

**Figure 4:**
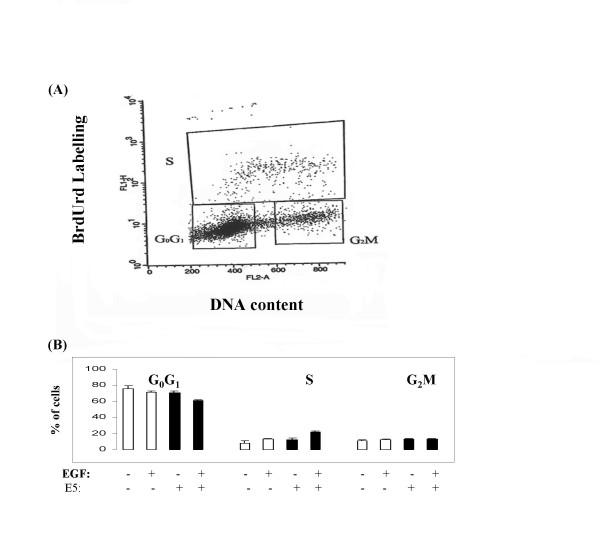
**Co-operative effects of HPV-16 E5 and EGF upon the cell-cycle progression**. **A: **Typical example of cell cycle analysis. **B: **Left to right: 'stop' control (S-phase = 8.25%); 'stop' control plus epidermal growth factor (EGF: S-phase = 13%); the reference isolate of HPV-16 (16: S-phase = 13.9%) and 16 with EGF (S-phase 37.6%). Results expressed as the mean and standard error of the mean for at least three independent measurements.

HPV-16 E5 co-operates with EGF to stimulate ^3^H-thymidine incorporation into DNA of human keratinocytes and murine fibroblasts [10]. Here we selected to analyse E5-induced mitosis by cell-cycle profiles as this may provide more detailed information than levels of ^3^H-thymidine incorporation alone (*e.g*. Figure 4A). To confirm the validity of this approach we determined whether there was a synergistic relationship between the reference sequence of HPV-16 E5 and EGF. Such a relationship between the EGF and HPV-16 E5 reference sequence was demonstrable (*e.g*. Figure 4B), in agreement with a previous study using a ^3^H-thymidine readout [10].

### E5 variants induce different cell-cycle profiles in the presence of EGF

**Figure 5:**
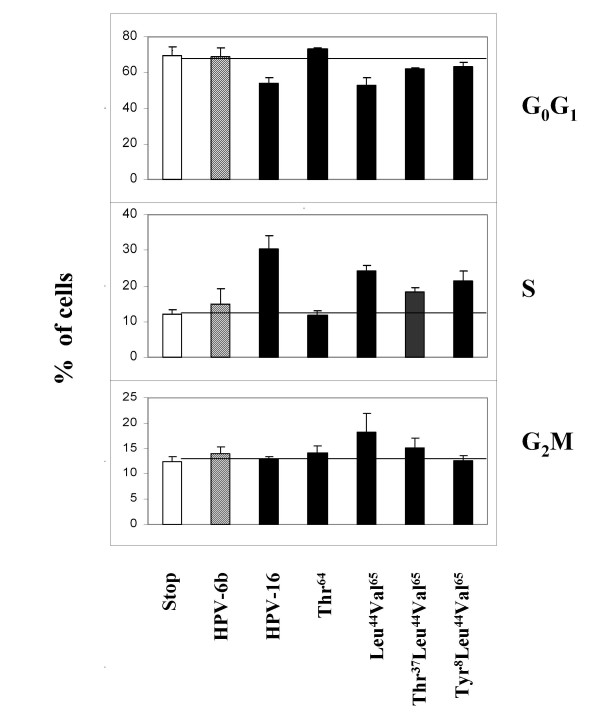
**Effects of E5 variants assayed with EGF upon cell-cycle profiles**. Results are expressed as the mean percentage (plus SEM) of cells in different stages of cell-cycling (G_0_G_1_, S & G_2_M) for each of HPV-16 E5 variant tested (solid bars), HPV-6b E5 (diagonal stripes), 'stop' constructs (open bars). Numbers of experiments: 'stop', n = 21; HPV-6b, n = 6; HPV-16, n = 9; Thr^64^, n = 9; Leu^44^Val^65^, n = 18; Thr^37 ^Leu^44^Val^65^, n = 6; and, Tyr^8^Leu^44^Val^65^, n = 11.

Compared to the 'stop' control, cells transfected with HPV-6b E5 or the HPV-16 E5 Thr^64 ^variant did not induce changes in G_0_G_1_-phase cell percentages (*versus *'stop', both p > 0.05: Figure 5). All other HPV-16 E5 variants caused reductions of G_0_G_1_-phase (all p < 0.05), this effect being greatest for Leu^44^Val^65 ^and the reference isolate (respectively, -16.2% & -15.5% *c.f*. 'stop'). Modest reductions in G_0_G_1_-phase were observed for the Tyr^8^Leu^44^Val^65 ^(-6.1%) and Thr^37^Leu^44^Val^65 ^(-7.4%) variants. The Thr^64 ^variant had a similar cell-cycle profile to the 'stop'.

Cells containing HPV-6b E5 or the HPV-16 E5 Thr^64 ^variant resembled the 'stop' in their S-phase profiles (both p > 0.05). Increases in S-phase were high for the reference isolate (+ 17.6% *c.f*. the 'stop') and for the Leu^44^Val^65 ^variant (+ 12.2%), but lower for Tyr^8^Leu^44^Val^65 ^(+9.4%) and for Thr^37^Leu^44^Val^65 ^(+ 6.3%: all p < 0.05). There were also increases in G_2_M-phase percentages for cells containing Leu^44^Val^65 ^(+ 5.8%: p < 0.05) or Thr^37^Leu^44^Val^65 ^(+ 2.8%: p > 0.05). Other E5 variants induced insignificant increases of G_2_M-phase percentages (Thr^64 ^+1.8%; HPV-6b E5, + 1.6%; reference isolate, + 0.6%; and, Tyr^8^Leu^44^Val^65^, + 0.2%: all p > 0.05).

### E5 variants with increased G_2_M-phase percentages exhibit increased cell-growth

**Figure 6:**
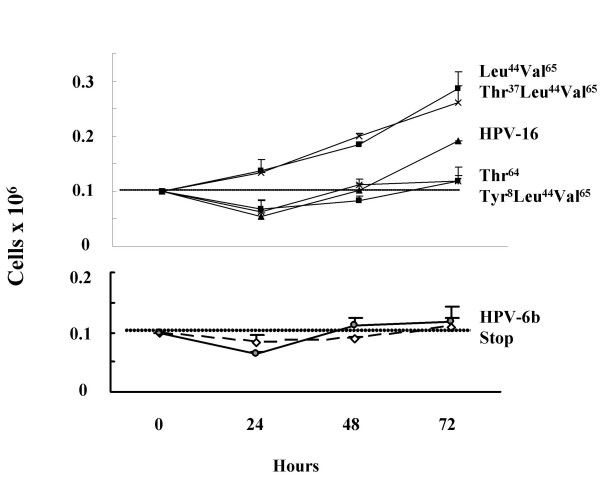
**Cell-growth curves**. Growth curves for cell lines stably transfected with different HPV-16 E5 variants, HPV-6b E5 or HPV-16 'stop'. Dotted lines indicate the initial seeding concentration, the lower graph was added for clarity. Error bars represent the standard error of the mean.

Static analyses of cell-cycle profiles can be difficult to interpret as the percentage values are inter-dependent variables. Thus, different cell populations could have identical cell-cycle profiles, but vastly dissimilar growth rates [27,28]. This problem was addressed by determining cell-growth at 24, 48 and 72 h in media containing EGF and minimal (2% v/v) serum supplement. Cells transfected with Leu^44^Val^65 ^and Thr^37^Leu^44^Val^65 ^variants exhibited most growth (Figure 6: both p < 0.02 *versus *the 'stop' at 72 h), whilst the HPV-16 E5 reference isolate induced a smaller increase in cell number (p < 0.05 *versus *the 'stop' at 72 h). Cells containing the Tyr^8^Leu^44^Val^65 ^and Thr^64 ^variants, or HPV-6b E5 grew slowly (all, p > 0.05 *versus *the 'stop' at 72 h). Alternate expression of cell-growth as cell-doubling times gave equivalent results (*e.g*. for: Leu^44^Val^65 ^mean doubling time = 56.6 h; Thr^37^Leu^44^Val^65 ^= 49 h; and, Tyr^8 ^Leu^44^Val^65^= 118 h).

### Tyr^8^substitution of HPV-16 E5 is associated with high levels of p21 protein

**Figure 7:**
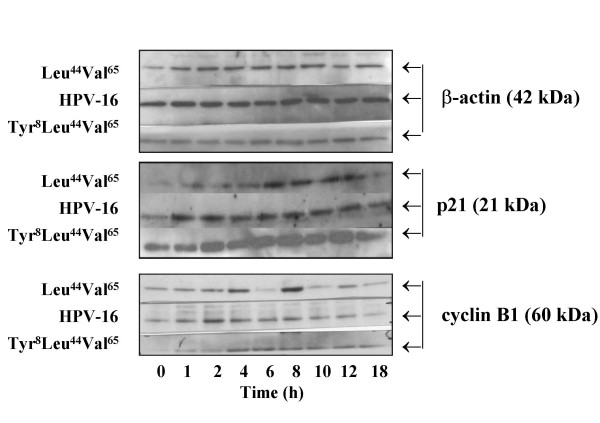
**Effect of different variants upon p21 and cyclin B expression**. Expression of p21 and cyclin B proteins over an 18 h period as determined in western blot experiments using β-actin levels as loading controls.

HPV-16 E5 can repress p21 transcription [29]. We thus determined levels of p21 and cyclin B1 proteins in cells containing the HPV-16 reference E5 sequence as well as a variant with a high-growth rate (Leu^44^Val^65^) and one with a low-growth rate (Tyr^8^Leu^44^Val^65^). Cells containing Tyr^8^Leu^44^Val^65 ^maintained high levels of p21 protein throughout the time course, whereas p21 was much lower in cells containing the reference isolate and, near undetectable for those containing Leu^44^Val^65 ^(Figure 7). There was also a delay before cyclin B1 could be detected in cells containing Tyr^8^Leu^44^Val^65 ^as compared to those stably transfected with the other two E5 proteins. These effects were not artefactual as levels of protein loaded were similar as indicated by the β-actin controls.

## Discussion

We demonstrate that the reference isolate of HPV-16 E5 can co-operate with EGF to reduce the percentage of cells in G_0_G_1_-phase, increase those in S-phase and increase cell-growth. HPV-6b E5 exhibited similar though much less marked changes than the HPV-16 E5 reference isolate, in agreement with a previous study [10]. In this report we have examined HPV-16 E5 variant activity under controlled conditions. Indeed, all were transcriptionally expressed under the same (T7) promoter and all had a minimal Kozak sequence [30] inserted around the initial ATG codon to insure equivalent translational efficiency. We have used these constructs to significantly extended previous observations observations on the biologic activity of HPV-16 E5 by analysing: HPV-16 E5 transcription in NIH-3T3 cells; cell-cycle progression; and, cell growth.

All HPV-16 E5 variants were translated in a cell-free wheat germ expression system equivalently, however, differences in E5 transcription were detected between stably transfected NIH-3T3 variant cell-lines. The HPV-16 E5 reference sequence and the Thr^64 ^variant were transcribed at much lower levels than the other variants. Analysis of cell-growth curves indicated that those with low levels of mRNA were also those which were slower-growing (HPV-16 reference and Thr^64^) and had lower percentages of cells in G_2_M phase. In contrast for those transcribed to similar levels in NIH-3T3 cells, the Leu^44^Val^65 ^and Thr^37^Leu^44^Val^65 ^variants exhibited high growth but, this was not the case for Tyr^8^Leu^44^Val^65^. These data suggest that whilst the addition of Thr^37 ^to the Leu^44^Val^65 ^backbone is neutral, addition of Tyr^8 ^is detrimental to cell-growth.

All HPV-16 E5 constructs – aside from Thr^64 ^variant – caused a fall in the percentage of cells in G_0_G_1_-phase and an increase in S-phase, indicating that these variant E5 proteins stimulate passage through the G_1_/S cell-cycle checkpoint. Such an increase in the proportion of cells in S-phase may be advantageous to HPV-16, permitting it to increase the number of viral copies *per *cell prior to division.

The HPV-16 E5 Leu^44^Val^65 ^and Thr^37^Leu^44^Val^65^variants, had greatest percentages of cells in G_2_M-phase and in greatest cell-growth. This biological activity may help explain why these particular E5 (*i.e*. RFLP pattern 2) variants are most prevalent (~70%) amongst wild populations of HPV-16 in inner-city London and most strongly associated with the presence of cervical lesions [24]. In contrast, the Tyr^8^Leu^44^Val^65 ^variant (which induced low cell growth) is an RFLP pattern 5 variant which is detected rarely amongst patients with lesions. Interestingly, the HPV-16 E5 RFLP pattern 2 HPV-16 E5 variants also co-segregate with nucleotide variation in the long control region that results in enhanced viral transcription *via *co-operation with the human POU transcription factor Brn3A [31].

The Leu^44 ^Val^65 ^substitutions may act to improve the structural integrity of E5 protein as both are α-helix stabilisers, whereas isoleucine (present at both sites in the reference isolate) is a helix destabiliser [32]. However, comparison of computer-predicted transmembrane regions [33] of the reference and Leu^44^Val^65 ^variant did not reveal significant differences (data not shown). Another possibility is that the Iso→Val^65 ^change may enhance the activity of two putative E5 functional domains: the voltage gating motif (^55^Ser-x-x-Arg-x-x-x-x-x-Iso-**Iso**^**65**^-Phe-Val-x-x-Pro-x-x-Leu-x-x-x-His^77^: [34]), present in the HPV-16 E5 reference isolate and all reported mammalian connexins; and, the proposed binding site for 16 kDa ATPase (aa 54–78: [2]). Conversely, another E5 variant with an adjacent change at position 64 (Thr^64 ^variant) exhibited an impoverished biological activity, confounding E5-induced mitogenesis at the G_0_/G_1 _checkpoint.

At least three EGF-independent E5 pathways exist (Figure 1), most notable being the transcriptional repression of p21 by E5 directly [35] or, indirectly, *via *E5 induction of *c-jun *[27,35]. The fact that cells containing the Tyr^8^Leu^44^Val^65 ^variant had the highest levels of p21 protein implies that the serine usually present at residue 8 may play an essential role in p21 repression. As the reference isolate and the Leu^44^Val^65 ^variant respectively exhibited an intermediate and low level of p21 expression it could also be inferred that residues 44 and 65 may also be involved in p21 repression.

Accumulation of high levels of p21 protein in cells containing the Tyr^8^Leu^44^Val^65 ^variant is also likely to effect cell-growth by up-regulating apoptosis. Indirect evidence for this was observed in the cell-growth assays: at 24 h numbers of Tyr^8^Leu^44^Val^65 ^containing cells had fallen to 67% of the seeding concentration (100%), in contrast those containing Leu^44^Val^65 ^variant increased to 137% (data not shown). These differences were even more marked at 48 h: Tyr^8^Leu^44^Val^65 ^cells were down to 54%, whereas cells containing Leu^44^Val^65 ^had nearly doubled (190%).

We also observed a reciprocal association between the levels of p21 and cyclin B1, this has been reported by others in several cell-systems and may represent a direct inhibitory effect of p21 upon cyclin-B1 biosynthesis [36,37]. Unlike the Tyr^8^Leu^44^Val^65 ^variant, insertion of a threonine (at position 64) into the Leu^44^Val^65 ^backbone appeared to have no marked effect.

## Conclusion

Using naturally-occurring amino acid sequence variants of HPV-16 E5 we have demonstrated that: (i) introduction of Thr^64 ^into the reference E5 sequence abrogates mitogenic activity most probably through low levels of transcription; (ii) combined substitution of Leu^44^Val^65 ^into the E5 reference backbone significantly enhances cell-cycle progression and cell-growth; (iii) addition of Thr^37 ^to the Leu^44^Val^65 ^variant had little effect upon mitogenic activity; and, (iv) inclusion of Tyr^8 ^into the Leu^44^Val^65 ^backbone severely inhibited E5 growth and E5 repression of p21 expression. These effects of HPV-16 E5 amino acid changes upon mitosis may – in part – help to explain why Leu^44^Val^65 ^HPV-16 E5 variants are most prevalent in 'wild' viral populations in the UK. Thus we suggest amino acids 8 and 64 are critical for the mitogenic activity of HPV-16 E5.

## Methods

### HPV samples

HPV-16 E5 variants isolated from clinical samples: Ted (Leu^44^Val^65^, EMBL accession number AJ244882), 9785 (Thr^37^Leu^44^Val^65^, AJ44863) and Twp3 (Thr^64^, AJ244840) [24] were studied. Permission for the collection of clinical specimens was provided by the Research Ethics Committee of St Thomas' Hospital. In addition, E5 DNA from: HPV-16 containing CaSki cells (Tyr^8^Leu^44^Val^65^, AJ24481); the reference isolate of HPV-16 [25,26]; and, from the low cancer-risk virus HPV-6b were investigated.

### Construction of recombinant DNA expression vectors

HPV-16 E5 genes were amplified in polymerase chain reactions (PCR) using *rTth*™ DNA polymerase in two separate reactions, so that E5 open reading frames (ORF) between nucleotides (nt) 3866 and 4077 (encoding E5 amino acids 6–76) were obtained. The first PCR utilised an upstream primer (^3836^GGAGCTAGCTCACCATGGCAAATCTTGATA^3865^) which included an artificial *Nhe*-1 cut site (underlined) and a minimal Kozak sequence [30] (^3847^ACCATGG^3853^) this motif was incorporated to promote equivalent translational efficiency for all constructs and introduces an artificial alanine residue at codon position two). The second PCR used a 5' primer (^3836^GGAGCTAG CTCACCATGGCATAACTTGATA^3865^) that also contained a 'stop' signal (underlined): both PCRs utilised the same downstream primer which encoded an artificial *Bam*H1 site (^4110^TACAGGATCCTTATG TAATTAAAAAGCGT GCATG^4078^). E5 PCR products were ligated into the *Nhe*1 and *Bam*H1 sites of *pc*DNA3.1*Myc*-His (Invitrogen Ltd.). The E5 open reading frame (ORF) of HPV-6b E5 was also PCR amplified (using the upstream primer: ^4103^TACTATATTGTTGCTAGCCCACCATGGTGCTAA^4135 ^and downstream, ^4366^TACAAATATAAAAAACGGGG ATCCCTAATTCATAT^4332^) and cloned using the same strategy. Plasmids were transformed into *E. Coli *JM109 cells and selected by ampicillin resistance. Positive colonies were screened by PCR and then sequenced to confirm the identity of the DNA inserts.

### In vitro translation

A cell-free wheat-germ expression assay (Promega Ltd.) was used to determine protein translation of T7 mRNA transcripts with individual reactions supplemented with 5 μl of ^35^S-labelled cysteine (1 Ci/l: Amersham International Ltd.). Radiolabelled cysteine was selected since all HPV-16 E5 variants contain 4 cysteine residues. Proteins were subjected to PAGE (below) and radioactivity detected by autoradiography.

### Preparation of stable NIH-3T3 cells expressing HPV E5 mRNA

NIH-3T3 cells (ATCC Ltd.) were maintained in Dulbecco's minimum essential medium supplemented with 40 mM L-glutamine, 2 × 10^6 ^U/l benzyl-penicillin, 2 g/l streptomycin sulphate (DMEM) and 10% (v/v) fetal calf serum (DMEM/FCS), in a humidified atmosphere of 5% (v/v) carbon dioxide in air. Cells were transfected with E5 containing plasmids using Lipofectin™ and grown in DMEM/FCS containing 1 g/l of G418 for 3 weeks (a dose cytotoxic for non-transfected NIH-3T3 cells within one week). Individual colonies of G418-selected cells were isolated and expanded in DMEM/FCS containing G418 to produce cell-lines for each variant. Individual cell-lines for each 'stop' construct were pooled. Variant cell-lines and the 'stop pool' were tested intermittently for mycoplasma infections: all were negative. Cell lines were tested for E5 mRNA expression using an adaption of the method described by Biswas *et al*. [5]. Briefly reverse transcriptase (RT) reactions were primed using the HPV-16 E5 downstream primer (^4110^TACAGGATCCTTATGTA ATTAAAAAGCGTGCAT^4078^) with Moloney murine leukemia virus reverse transcriptase then samples were subjected to a nested PCR using internal primers located within the E5 ORF [5].

### Cell-cycle analyses

NIH-3T3 cells transfected with HPV-16 variants, HPV-16 'stop', or HPV-6b were seeded at 0.1 × 10^6 ^cells in 5 ml of DMEM/FCS (containing no G418) into 25 cm^3 ^flasks. Cells were serum-starved for 24 h in DMEM, media was then replaced with fresh DMEM with (or without) recombinant human EGF (20 μg/l: Boehringer-Mannheim Ltd.) and cells incubated for a further 24 h. Bromo-deoxyuridine (BrdUrd: 10 μM in DMEM) were added and cells washed twice by centrifugation through 10 ml of Dulbecco's phosphate buffered saline (PBS) for 5 min at 200 g at room temperature (rt) and then fixed with 2 ml of ice-cold aqueuous 70% (v/v) ethanol. Fixative was removed and cells incubated in 1 ml of 0.1 M HCl containing 1 g/l pepsin for 12 min at 37°C. Reactions were halted by centrifugation (as above) through, and re-suspension in, PBS. Murine monoclonal anti-BrdUrd IgG_1 _heavy and kappa light chains (Becton-Dickenson Ltd: 250 μl per litre of PBS which contained 0.5% [v/v] Tween-20™ and 1% [v/v] FCS) was added for 1 h at rt, cells were washed with PBS and then incubated for 30 min at rt in the dark with fluorosceine-isothiocyanate (FITC) labelled Fab_2 _fragments of rabbit anti-mouse immunoglobulin (Dako Ltd.) at rt. After a further wash in PBS cells were re-suspended in, and stained with, 1 ml of propidium iodide/RNAse solution (50 g propidium iodide and 200 g RNAse/l PBS) for 15 min at rt before analysis on a Becton-Dickenson flow cytometer (FACSCalibur™). Data from 10,000 events (*i.e*. stained cells) were analysed (for a minimum of four times) for each reading after separation of single intact nuclei from debris and cell-clumps by gating on an FL2 area/width plot. Cells were then characterised on the basis of detection of green (FITC) and red (propidium iodide) fluorescence.

### Cell-growth

Cells were grown in 2% (v/v) in DMEM containing 2% (v/v) fetal calf serum, supplemented with EGF (20 μg/l), after seeding at a density of 0.1 × 10^6 ^cells in 20 cm^3 ^Petri dishes. Cells were fed daily with fresh media containing EGF and cultures harvested at 24, 48 and 72 h by detachment from Petri dishes by exposure to Versene (5 ml for 2 min at 37°C). One millilitre of DMEM/FCS was added to inactivate the trypsin then cells were pelleted by centrifugation (200 g for 15 min at rt). Cell pellets were re-suspended in DMEM/FCS and viable cell numbers determined immediately by trypan-blue exclusion staining [38].

### Western blots

NIH-3T3 cells (0.1 × 10^6 ^cells in 5 ml of DMEM/FCS containing no G418) into 20 cm^3 ^Petri dishes and left overnight. Cells were serum-starved for 24 h in DMEM, grown in DMEM supplemented with EGF (as above), then harvested over an 18 h period. Cells were lysed by suspension in RIPA buffer and cellular debris removed by centrifugation at 10,000 g for 30 sec at rt. Protein concentrations were determined using a commercial assay (BioRad Ltd.) and adjusted to enable 5 μg of total protein to be added to each well of a PAGE gel. Cellular proteins were subjected to PAGE under reducing conditions through a 4–12% Tris/glycine gradient gel (Novex Ltd.) and blotted onto Hybond-P™ membranes (Amersham International Ltd.). Each blot was next cut into strips containing the proteins of interest: cyclin B1 (60 kDa), β-actin (42 kDa) and p21 (21 kDa). Strips were blocked overnight at rt using 5% (w/v) dried milk powder (Marvel™, Premier Brands UK Ltd.) in Tris-buffered saline/Tween™ (20 mM Tris [hydroxymethyl]-amino methane; 0.2 M sodium chloride and 0.1% [v/v] Tween-20™, pH 7.5: TBS-T).

Blot strips were washed three times with TBS-T then incubated for 1 h at rt with rabbit anti-β-actin (1/1000 dilution), mouse anti-p21 (2.5 mg/l) or, mouse anti-cyclin B1 (2 mg/l: Pharminogen Ltd.) antibodies. After two washes in TBS-T strips were immersed in the appropriate horse-radish peroxidase-labelled secondary antibody (sheep anti-mouse immunoglobulin or donkey anti-rabbit immunoglobulin: Amersham International Ltd. each at a dilution of 1/250) for 1 h at rt. Bound antibody was detected using an ECL-plus™ chemiluminescence kit and ECL Hyperfilm™ (Amersham International Ltd.).

### Statistical analyses

Students' t-tests were used to assist the interpretation of data.

## Abbreviations

ATCC, American type culture collection; BrdUrd, Bromo-deoxyuridine; DMEM, Dulbecco's minimal essential medium; EGF, epidermal growth factor; EGFr, epidermal growth factor receptor; EMBL, European molecular biology laboratory; FACS, Fluorescence activated cell sorting; FCS, fetal calf serum; FITC, Fourosceine isothiocyanate; HPV, human papillomavirus; KDa, Kilo Daltons; ORF, Open reading frame; PAGE, Polyacrylamide gel electrophoresis; PBS, Dulbecco's phosphate buffered saline; PCR, Polymerase chain reaction; RIPA, Radio-immunoprecipitation assay; rt, Room temperature; RT, Reverse transcriptase;

*rtTh*, Recombinant thermostable Thermus thermophilus DNA polymerase; TBS, Tris buffered saline; TBS-T, TBS-Tween ™; UK, United Kingdom.

## Competing interests

The author(s) declare that they have no competing interests.

## Authors' contributions

RN prepared samples, PCR amplifications and DNA sequencing. CM designed and perfected the PCRs and assisted in data analyses. BK oversaw the molecular biological approach to this project and assisted with data analyses. JC devised the research and assisted with the data analyses. JMB directed the cell-cloning, cell-growth and cell-cycle analyses.
